# Metal Dealing at the Origin of the Chordata Phylum: The Metallothionein System and Metal Overload Response in *Amphioxus*

**DOI:** 10.1371/journal.pone.0043299

**Published:** 2012-08-14

**Authors:** Maria Guirola, Sílvia Pérez-Rafael, Mercè Capdevila, Òscar Palacios, Sílvia Atrian

**Affiliations:** 1 Departament de Genètica, Facultat de Biologia, Universitat de Barcelona, Barcelona, Spain; 2 Departament de Química, Facultat de Ciències, Universitat Autònoma de Barcelona, Barcelona, Spain; Queen Mary University of London, United Kingdom

## Abstract

Non-vertebrate chordates, specifically amphioxus, are considered of the utmost interest for gaining insight into the evolutionary trends, i.e. differentiation and specialization, of gene/protein systems. In this work, MTs (metallothioneins), the most important metal binding proteins, are characterized for the first time in the cephalochordate subphylum at both gene and protein level, together with the main features defining the amphioxus response to cadmium and copper overload. Two MT genes (*BfMT1* and *BfMT2*) have been identified in a contiguous region of the genome, as well as several ARE (antioxidant response element) and MRE (metal response element) located upstream the transcribed region. Their corresponding cDNAs exhibit identical sequence in the two lancelet species (*B. floridae* and *B. lanceolatum*), *BfMT2* cDNA resulting from an alternative splicing event. BfMT1 is a polyvalent metal binding peptide that coordinates any of the studied metal ions (Zn, Cd or Cu) rendering complexes stable enough to last in physiological environments, which is fully concordant with the constitutive expression of its gene, and therefore, with a metal homeostasis housekeeping role. On the contrary, BfMT2 exhibits a clear ability to coordinate Cd(II) ions, while it is absolutely unable to fold into stable Cu (I) complexes, even as mixed species. This identifies it as an essential detoxification agent, which is consequently only induced in emergency situations. The cephalochordate MTs are not directly related to vertebrate MTs, neither by gene structure, protein similarity nor metal-binding behavior of the encoded peptides. The closest relative is the echinoderm MT, which confirm proposed phylogenetic relationships between these two groups. The current findings support the existence in most organisms of two types of MTs as for their metal binding preferences, devoted to different biological functions: multivalent MTs for housekeeping roles, and specialized MTs that evolve either as Cd-thioneins or Cu-thioneins, according to the ecophysiological needs of each kind of organisms.

## Introduction

Metallothioneins (MTs) constitute a heterogeneous superfamily of ubiquitously occurring, low molecular weight, cysteine rich proteins, which coordinate divalent (Zn^2+^, Cd^2+^) or monovalent (Cu^+^) metal ions through metal-thiolate bonds that impose a definite polypeptide folding (see [1], [2] for recent revisions). No single biological role has been assigned to these peptides, but, instead, several functions have been proposed [3], ranging from toxic metal protection to physiological metal homeostasis, and also including free radical scavenging, oxidative stress protection, antiapoptotic defense and control of the redox status of the cell. Another fascinating *black hole* in knowledge of MT is their origin and functional differentiation through evolution. Although a polyphyletic origin has been proposed [4], their evolutionary history is particularly hard to interpret by means of standard molecular evolution criteria.

Vertebrate MTs are grouped in the *Family 1* of the taxonomy-based Binz and Kägi′s MT classification [5], available at www.bioc.unizh.ch/mtpage/classif.html, therefore including all the isoforms identified in mammals, birds, reptiles, amphibians and fishes. These MTs are 60- to 68- amino acid long polypeptides, encompassing 20 cysteines (19 of which are totally conserved). They fold into two structural domains upon divalent metal ion coordination, namely the ß-domain (N-terminal moiety) and the α–domain (C-terminal moiety), connected by a hinge with a low number of residues. Vertebrate MT encoding genes are composed of three exons and two introns of variable lengths, but interrupting the coding regions at conserved positions. MT polymorphism is constant from fishes to mammals, which exhibit a four-member cluster (MT1 to MT4) [6], with differences in gene (*i.e*. gene expression pattern) and protein level (*i.e*. isoform metal binding preferences) [7]. Avian MTs, the closest mammalian relatives, exhibit less polymorphism, with two isoforms identified in chicken [8]. Their genes share the same exon/intron structure as mammalian *MTs*, and they are regulated by similar stimuli including metal overdose and oxidative stress [8], [9], [10]. CkMT1 (chicken MT1) isoform is able to bind divalent and monovalent metal ions with an intermediate affinity between those observed for mammalian MT1 (classified as a Zn-thionein) and MT4 (Cu-thionein) [9]. All the fish MT genes and proteins reported up to date also share the same structural and functional features described for mammalian MTs [11]. A single MT has been described so far in amphibians, with a gene structure more similar to avian than to mammals and whose expression is inducible by zinc, copper and cadmium [12]. Finally, little information is available regarding the MT system in reptiles, although some isoforms have been identified that bear a strong similarity to avian MTs, including preservation of cysteine alignment and structure [13]. All this information has drawn a fairly clear picture of the evolution of the MT system inside the vertebrate subphyla (*cf*. http://www.bioc.unizh.ch/mtpage/trees.html), but there is still no clue about the point (or points) of origin of MT molecular diversification of vertebrate MTs regarding other organism groups. Besides vertebrates, the chordata phylum comprises two further subphyla: cephalochordates (the lancelets or amphioxus) and tunicates (previously urochordates, the sea-squints). The privileged position of these groups in the *tree of life* has been highly relevant for their use in studies of the molecular phylogeny and genome evolution of chordate/vertebrate organisms [14], [15], [16]. Hence, the study of genome evolution from the two prochordate groups to chordates revealed a final quadruplication in vertebrates, but most of the resulting duplicated genes were apparently lost and severe rearrangements, worsened by a high number of transposition events, are likely to blur the evolutionary transition to vertebrates [17]. In fact, the situation is so unclear that the relationship between the prochordate taxa and vertebrates is still a matter of much debate [18].

**Figure 1:**
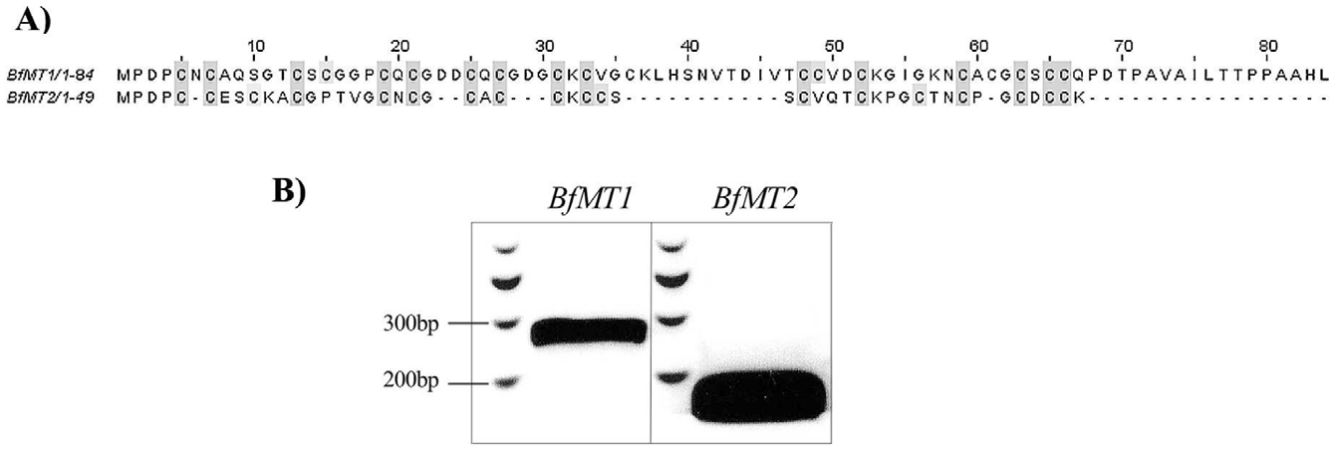
Protein sequences deduced of both identified Amphioxus metallothionein genes, which are patently transcribed in adult organisms. (**A**) Aligned protein sequences of the two identified amphioxus MTs, BfMT1 and BfMT2, *in silico* translated from the rbfeg037o01 and bfne062f22 cDNA clones, respectively. Cysteine residues are highlighted in gray. Alignment was performed using the T-Coffee software. (**B**) RT-PCR amplification of BfMT1 and BfMT2 cDNA using adult *B. lanceolatum* total mRNA as a template. Both band sizes corresponded to that expected for the respective coding sequences (255 bp for BfMT1 and 150 bp for BfMT2). The amplified cDNAs were cloned, and their sequences were verified as exact matches with respect to the *B. floridae* library cDNA clones.

**Figure 2:**
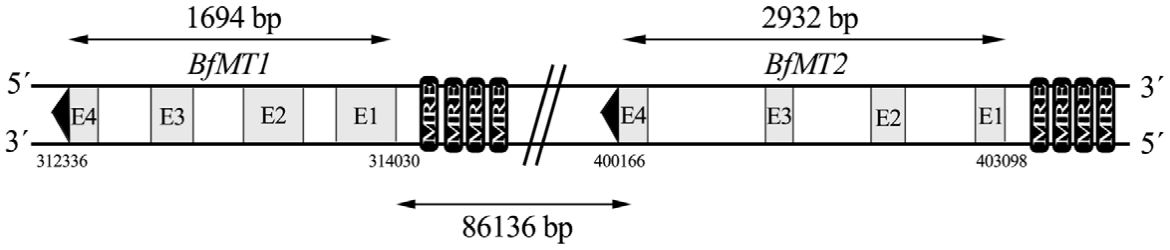
The genomic localization, orientation and structure of the *BfMT1* and *BfMT2* genes coincide in a 100-Kb region of the *B. floridae* genome. Both are located in the negative strand of the *B. floridae* genome scaffold 398. Exact location in pair bases with respect to the scaffold sequence is represented below each gene. The distance between both genes, as well as their size, are also indicated. Exons are shown in gray, and numbered E1 to E4. Black arrows indicate the transcription direction and the metal response elements (MREs) identified in the promoter regions are represented as black boxes.

**Figure 3:**
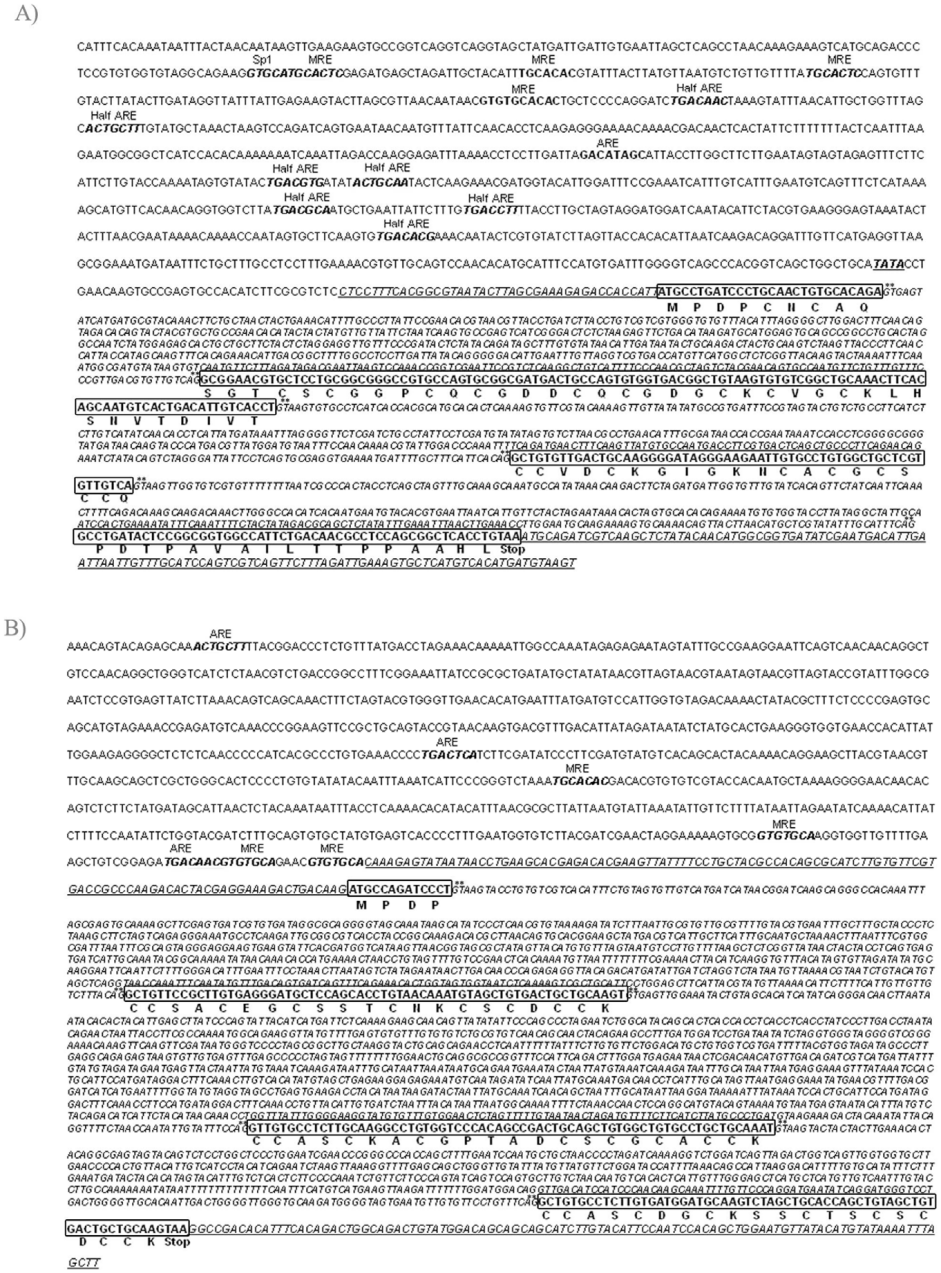
The analysis of the *BfMT1* and *BfMT2* genomic sequences identify all the elements of these genes. Promoter regions (-1 kb) are included and the *in silico* identified regulatory sequences (MRE and ARE) are highlighted in bold. 5′and 3′UTR regions, corresponding to the cDNA sequences of the cDNA clones, are included and represented in italics and underlined. Coding sequences are boxed in their corresponding exons and translated protein sequences are included below each exon.

**Figure 4:**
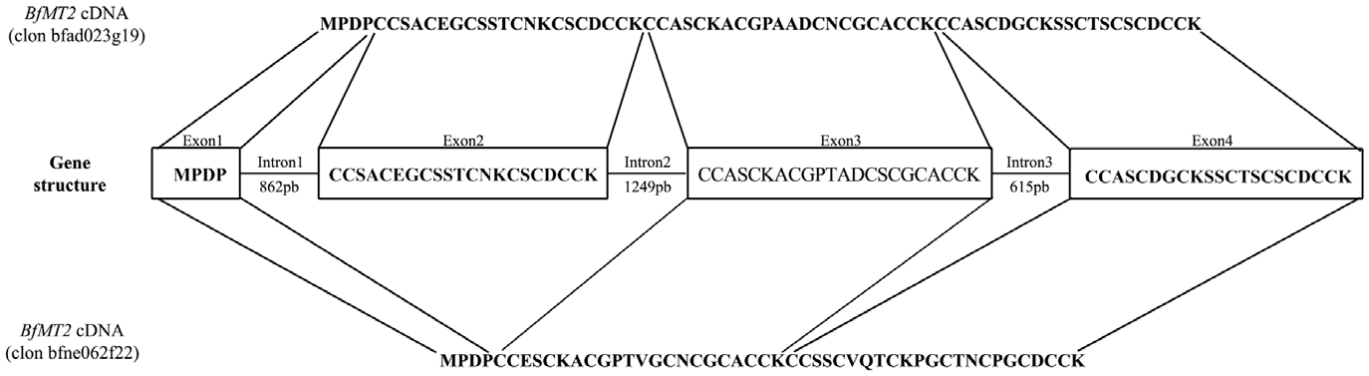
*BfMT2* transcription proceeds through alternative splicing patterns. Schematic representation of the *BfMT2* gene structure and the protein sequence corresponding to each exon (in the centre), and the protein sequences for each alternative cDNA. *BfMT2* coding sequence used for translation was extracted from the NCBI Databank and corresponds to *B. floridae* strain S238N-H82. cDNA clone sequences were extracted and obtained from the *B. floridae* cDNA Database.

**Figure 5:**
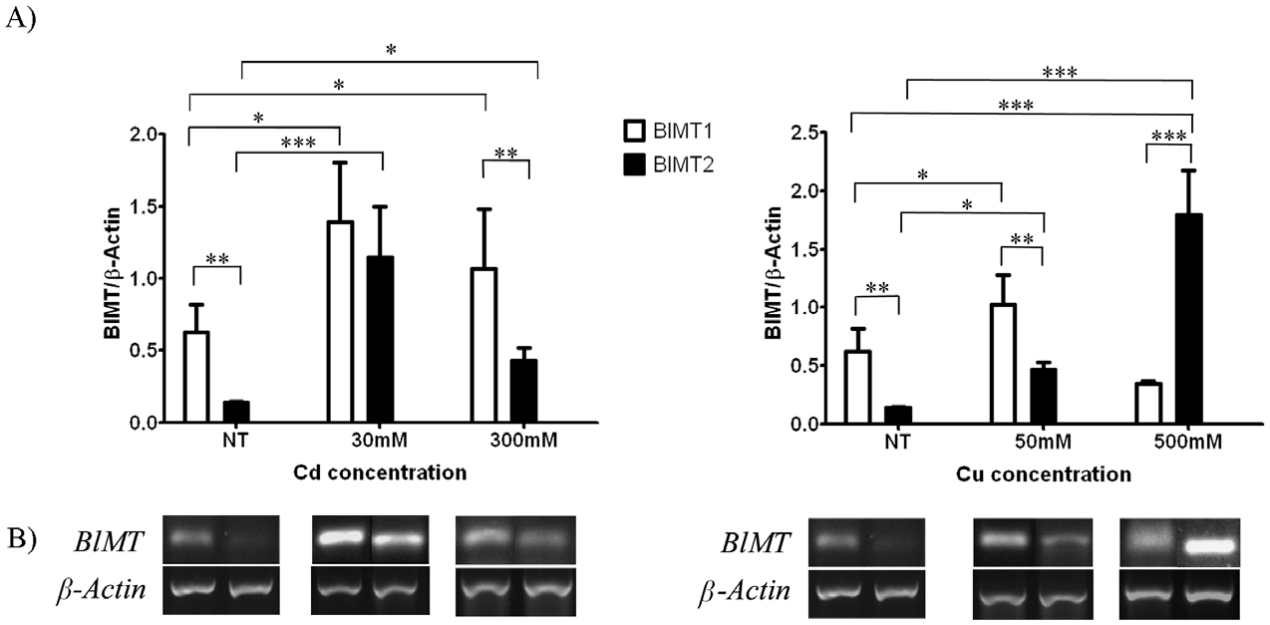
*BlMT1* is essentially constitutive, while *BlMT2* is an inducible gene, as revealed in metal and non-metal treated *B. lanceolatum* organisms. (**A**) Semi quantification by RT-PCR of transcription rates under Cu and Cd supplementation, and statistical comparison. Data for each gene and condition is normalized by the corresponding value for the constitutive gene *β-actin*, previously homogenized between organisms and groups. Data represent mean and standard deviation of six organisms. Significance was assessed using the Newman-Keuls statistical test. Stars denote statistical significance as follows: * as equivalent to a significance of P<0.05, ** equivalent to P<0.01, and *** to P<0.001. B) Best examples of RT-PCR bands representing the results observed in A). NT stands for non-treated organisms.

**Figure 6:**
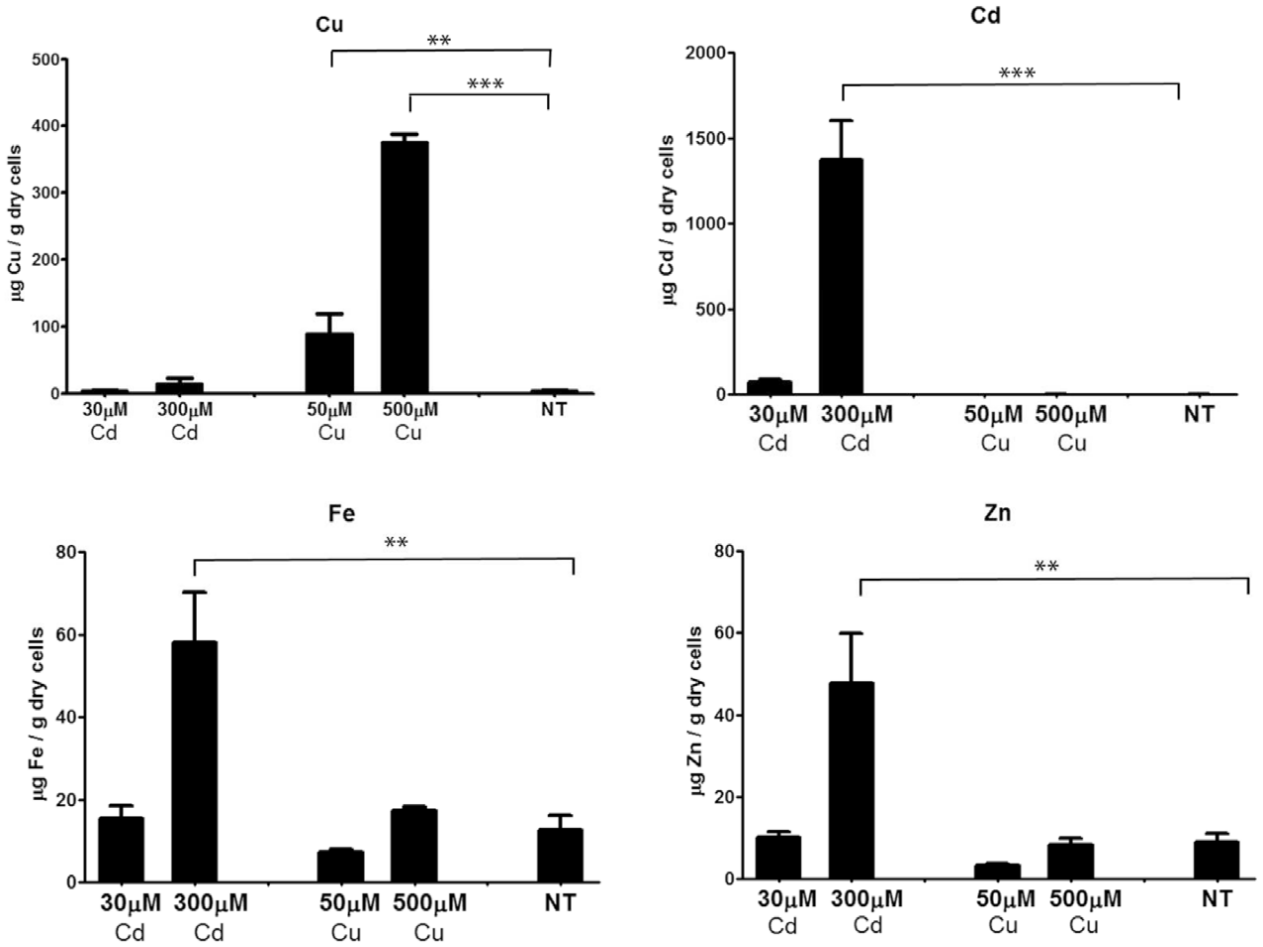
ICP-measured Cd and Cu accumulation in treated lancelets follow different patterns. *B. lanceolatum* organisms were treated with two different concentrations of Cd or Cu, or non-metal treated (NT). Data is represented as mean and standard deviation of six organisms per group. Statistical significance is represented as ** equivalent to P<0.01, and *** to P<0.001 according to the Newman-Keuls statistical test.

**Figure 7:**
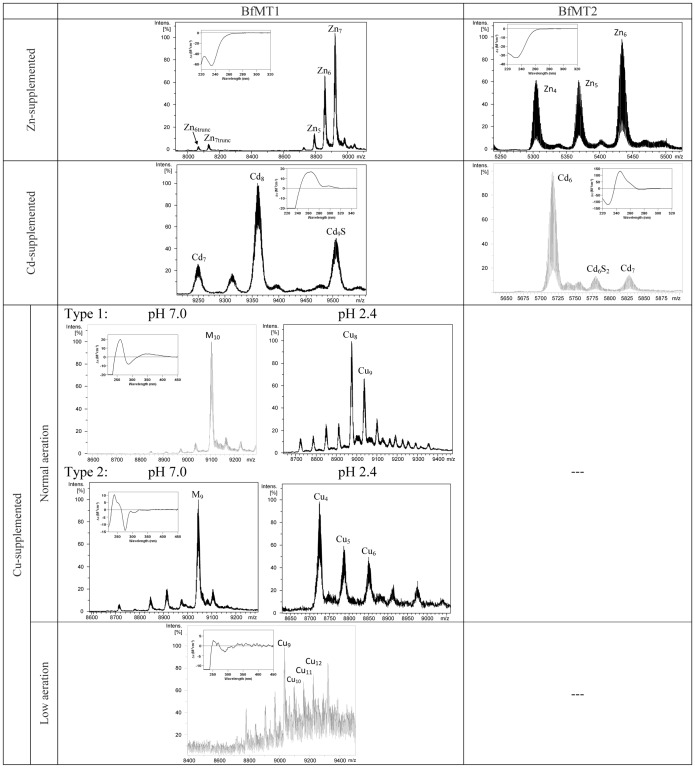
The BfMT1 and BfMT2 peptides exhibit different metal binding abilities. Deconvoluted ESI-MS spectra, at neutral pH, corresponding to the BfMT1 and BfMT2 recombinant syntheses, obtained from bacterial cultures grown in Zn-, Cd- and Cu-supplemented. Insets correspond to the CD fingerprint of each preparation. The ESI-MS spectra at pH 2.4 is also included for the Cu-BfMT preparations obtained under normal aeration conditions of the cultures.

**Table 1:** Analytical characterization of the recombinant preparations of the Zn-, Cd- and Cu-complexes yielded by BfMT1 and BfMT2.

	Zn	Cd	Cu	
			Normal aeration	Low aeration
**BfMT1**			Type 1:	
		2.6 10^−4^ M	0.57 10^−4^ M	0.19 10^−4^ M
		(1.0 10^−4^ M)	2.7 Zn/MT	11.9 Cu/MT
	1.7 10^−4^ M		5.1 Cu/MT	
	5.8 Zn/MT		Type 2:	
		4.4 Cd/MT	0.55 10^−4^ M	
		(10.3 Cd/MT)	1.8 Zn/MT	
			8.0 Cu/MT	
**BfMT2**		2.4 10^−4^ M	–	–
	2.6 10^−4^ M	(0.9 10^−4^ M)	–	–
	5.0 Zn/MT	3.3 Cd/MT	–	–
		(7.6 Cd/MT)	–	–

In all cases the Zn, Cd, Cu and S content was measured by ICP-AES but only detectable contents are shown. The protein concentration values and Cd/MT ratios shown in parenthesis correspond to those measured by acid ICP-AES.

**Figure 8:**
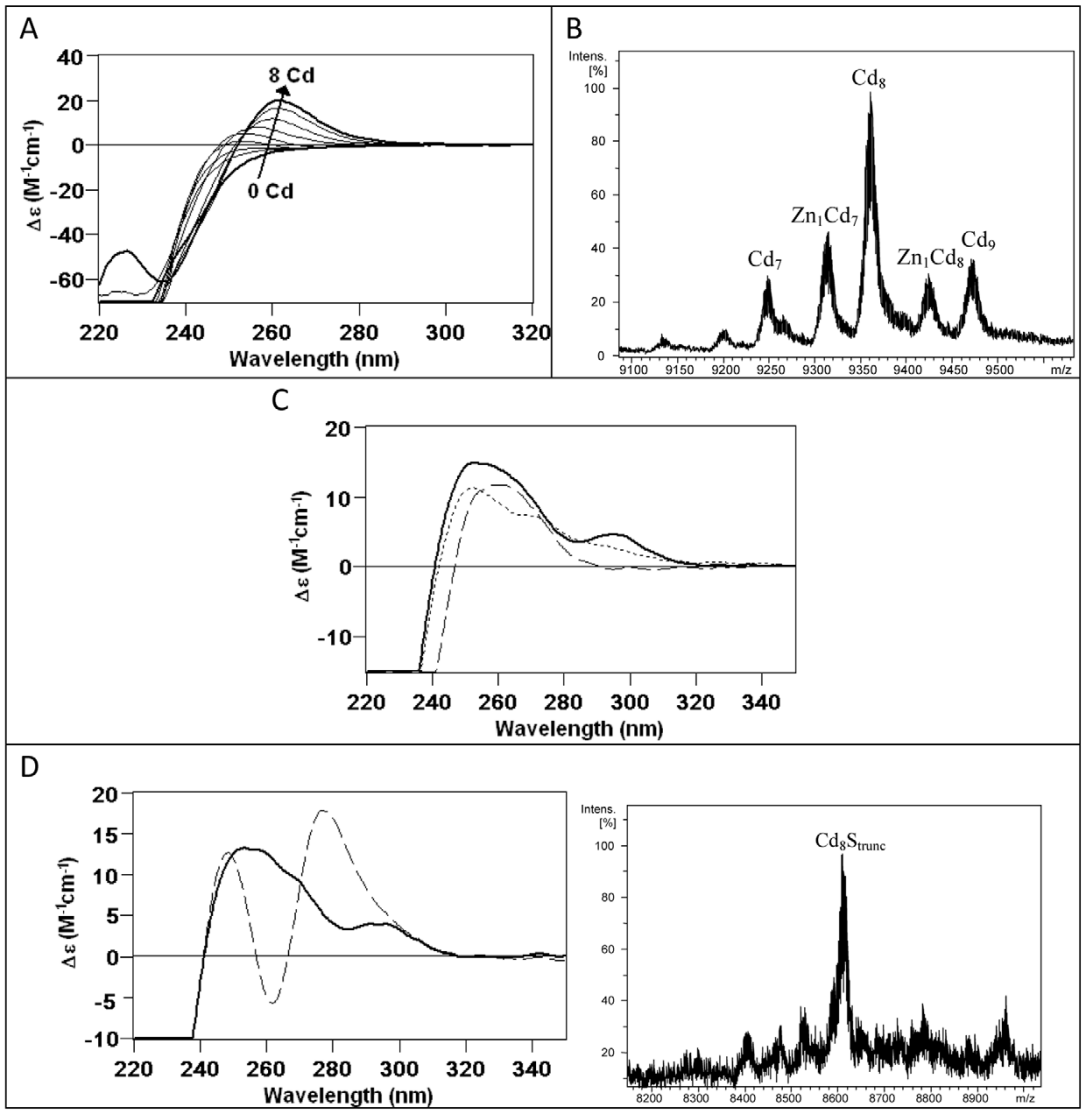
The *in vitro* Cd(II) binding abilities of BfMT1 points to a partial preference for divalent metal ion coordination. (A) CD corresponding to the titration of a 10 µM solution of Zn-BfMT1 with Cd(II) at pH 7.0 until 8 Cd(II) eq added (B) Deconvoluted ESI-MS spectrum of an aliquot corresponding to the addition of 8 Cd(II) eq to Zn-BfMT1. (C) Comparison of the CD spectra of the recombinant Cd-BfMT1 preparation (solid line), the acidified and reneutralized sample (dashed line), and the latter after the addition of 4 S^2−^ equivalents (dotted line). (D) Comparison of the CD spectra of the Cd-BfMT1 sample before (solid line) and after (dashed line) 28-days of evolution under inert atmosphere and ESI-MS spectrum of the final sample.

**Figure 9:**
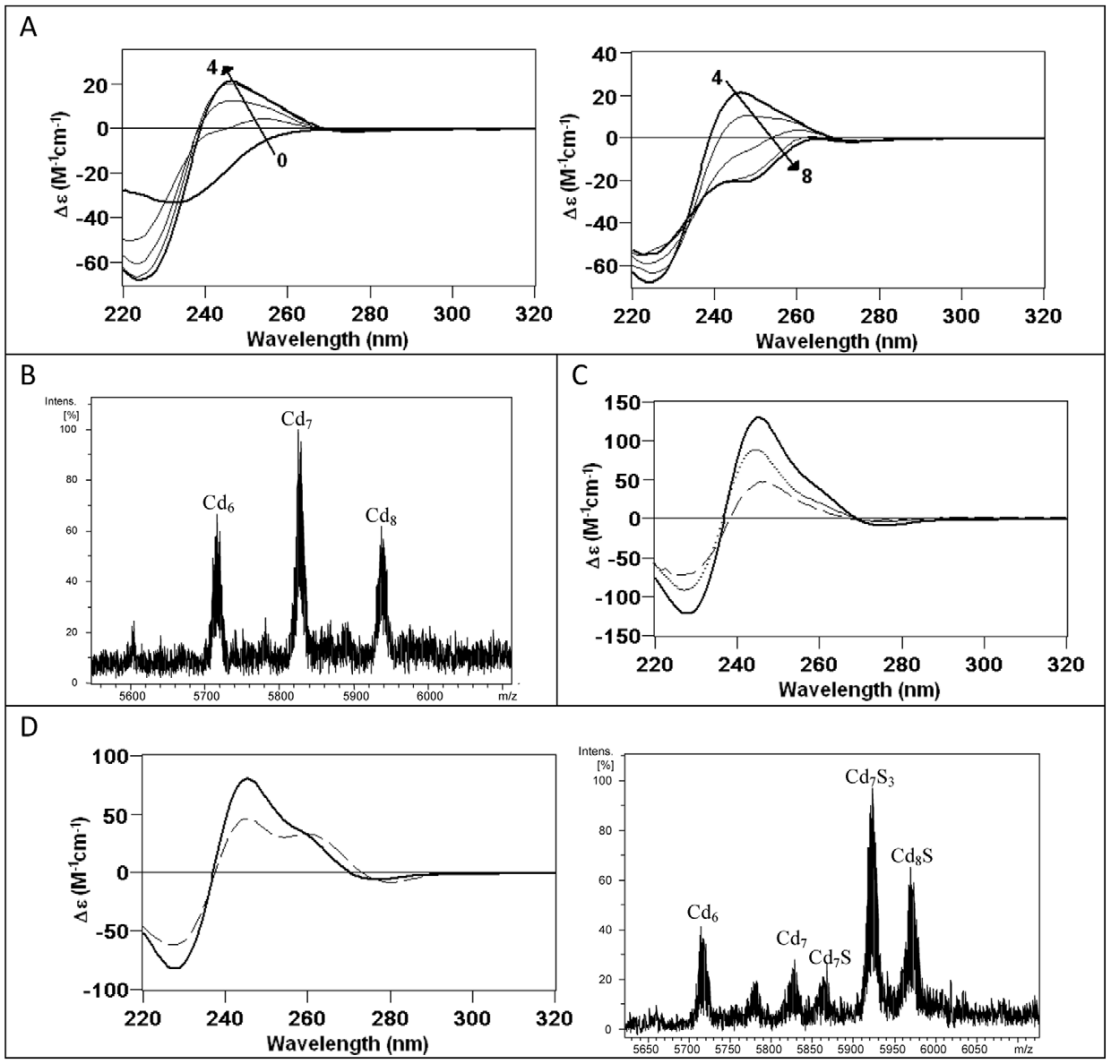
The *in vitro* Cd(II) binding abilities of BfMT2 points to a partial preference for divalent metal ion coordination. (A) CD corresponding to the titration of a 10 µM solution of Zn-BfMT2 with Cd(II) at pH 7.0 from 0 to 4 and from 4 to 8 Cd(II) eq added. (B) Deconvoluted ESI-MS spectrum of an aliquot corresponding to the addition of 8 Cd(II) eq to Zn-BfMT2. (C) Comparison of the CD spectra of the recombinant Cd-BfMT2 preparation (solid line), the acidified and reneutralized sample (dashed line), and the latter after the addition of 4 sulfide equivalents (dotted line). (D) Comparison of the CD spectra of the Cd-BfMT2 sample before (solid line) and after (dashed line) 28-days of evolution under inert atmosphere and ESI-MS spectrum of the final sample.

**Figure 10:**
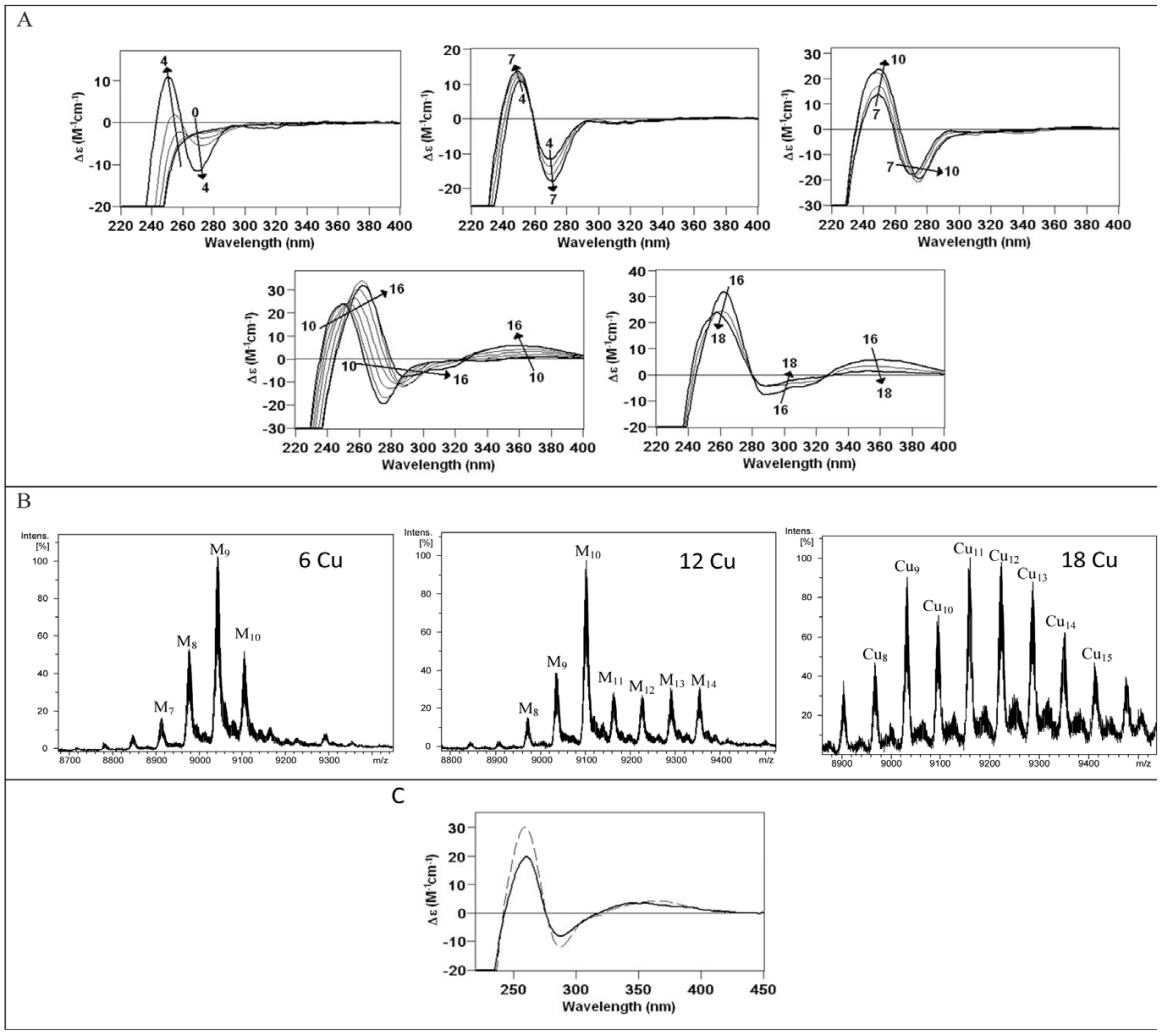
The *in vitro* Cu(I) binding abilities of BfMT peptides shows the preference of BfMT1 *vs*. BfMT2 to coordinate this metal ion. (A) CD corresponding to the titration of a 10 µM solution of Zn-BfMT1 with Cu(I) at pH 7.0 until 18 Cu(I) eq added. The arrows show the number of Cu(I) eq added at each stage of the titration. (B) Deconvoluted ESI-MS spectrum of several aliquots extracted from the solution at 6, 12, and 18 Cu(I) eq added to Zn-BfMT1. (C) Comparison of the CD spectra of the recombinant Cu-BfMT2 preparation (solid line), and that of the Zn-BfMT1 sample after the addition of 12 Cu(I) eq (dashed line).

**Figure 11:**
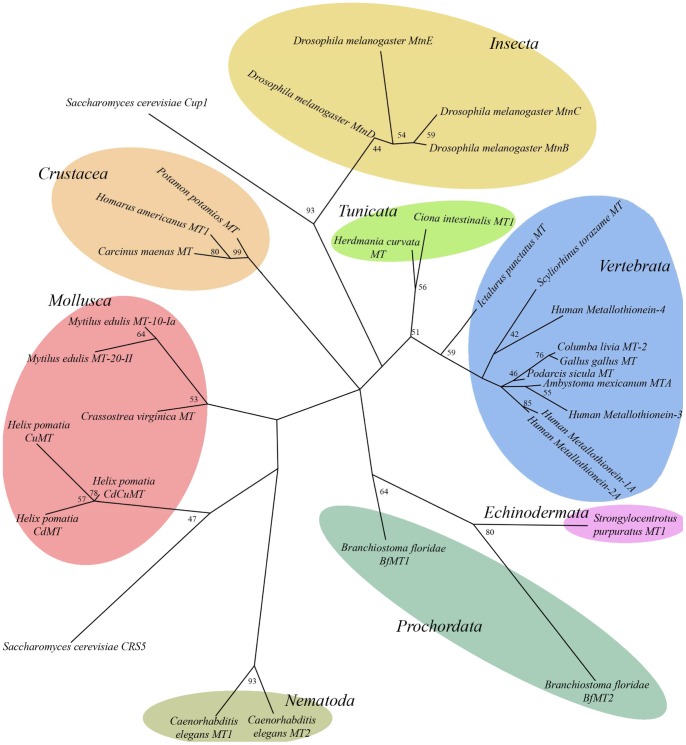
Protein distance analysis of MTs from organisms of different taxa positions *Branchiostoma* MTs at the base of vertebrates and close to Echinoderm MTs. The Maximum Likelihood tree was obtained by the Phylip method, and the corresponding bootstrap values (in percentages) obtained are included. Sequences and their Uniprot IDs are: Human MT-1A (P04731), Human MT-2A (P02795), Human MT-3 (P25713), Human MT-4 (P47944), *Gallus gallus* MT (P68497), *Columba livia* MT-2 (P15787), *Podarcis sicula* MT (Q708T3), *Ictalurus punctatus* MT (O93571), *Scyliorhinus torazame* MT (Q6J1T3), *Ambystoma mexicanum* MTA (O42152), *Mytilus edulis* 10 Ia (P80246), *Crassostrea virginica* MT (P23038), *Mytilus edulis* MT-20-II (P80252), *Helix pomatia* CdCuMT (D1LZJ8), *Helix pomatia* CdMT (P33187), *Helix pomatia* CuMT (P55947), *Homarus americanus* MT1 (P29499), *Carcinus maenas* MT (P55948), *Potamon potamios* MT (P55952), *Drosophila melanogaster* MtnB (P11956), *Drosophila melanogaster* MtnC (Q9VDN2), *Drosophila melanogaster* MtnD (Q8I9B4), *Caenorhabditis elegans* MT1 (P17511), *Caenorhabditis elegans* MT2 (P17512), *Saccharomyces cerevisiae* Cup1 (P0CX80), *Saccharomyces cerevisiae* CRS5 (P41902). The following sequences were obtained from GeneBank: *Strongylocentrotus purpuratus* MT1 (AAA30061.1), *Ciona intestinalis* MT1 (ACN32211.2), *Herdmania curvata* MT (AY314949.1). *Drosophila melanogaster* MtnE sequence was obtained from FlyBase (FlyBaseID: FBpp0293071).

Precisely regarding the MT system in non-vertebrate chordates, very little information is available at present. Among tunicates, two mRNA from *Herdmania curvata* are annotated as MT-like in Gene Bank (AY314949 and AY314939 accession numbers), and more recently an MT gene (*CiMT-1*) has been characterized in *Ciona intestinalis* [19]. Like vertebrate MTs, *CiMT-1* is composed of three exons and two introns, but only two of its exons comprise coding stretches, so that the encoded MT peptide is the shortest currently known in deuterostomes. The corresponding protein still retains the characteristic 30% cysteine content, although showing very little similarity with other reported MTs. Exposure to cadmium induces the expression of CiMT-1, but the presence of various putative response elements in the promoter region suggests that the transcription of the gene could be activated by other stimuli [19]. For cephalochordates, no information was available on any MT aspect at the beginning of this study, except for an annotated ORF in amphioxus (*Branchiostoma floridae*), predicted as a putative MT.

Since we consider that the knowledge of non-vertebrate chordates, specifically of amphioxus, is of the outmost interest for understanding and gaining insight into the evolutionary trends of the MT proteins, we have undertaken a study of the lancelet MT system, exploiting the recently completed amphioxus genome information [16]. Hence, we here report the first identification and characterization of two different amphioxus MT genes/proteins (BfMT1 and BfMT2), including the characterization of the gene structure and their metal induction pattern, the metal binding behavior of the encoded peptides and also the first characterization of the response of lancelets to metal intoxication. *BfMT1* appears to be an essentially constitutive gene, *BfMT2* is a clear inducible gene. The consideration of the Cd and Cu transcription regulation trends for the two genes, as well as of the metal binding behavior of both recombinantly synthesized peptides converge in the hypothesis that the serve very different purposes in the amphioxus organisms. BfMT1 is a polyvalent metal binding peptide, able to coordinate any of the studied metal ions (Zn, Cd or Cu), rendering complexes stable enough to last in physiological environments, which is fully concordant with the constitutive character of its encoding gene, and therefore, with a metal homeostasis housekeeping role. On the contrary, BfMT2 exhibits an clear ability to coordinate Cd(II) ions, while it is absolutely unable to fold into stable complexes under Cu(I) surplus, even as mixed Zn, Cu species. This is compatible with an essential cadmium detoxification role, which is consequently only induced in emergency situations. Notably, the Cd and Cu internal accumulation in metal treated organism fully confirms this metal binding behavior. A comprehensive consideration of all their features suggests interesting hypotheses not only for MT differentiation and specialization through evolution, but also for the phylogenetic relationship between cephalochordates and their surrounding taxa.

## Materials and Methods

### Bioinformatics methods for identification and analysis of MT genes in the amphioxus genome

Accession to the Florida lancelet species (*Branchiostoma floridae*) genome data (v1.0) was via the corresponding platform of the DOE Joint Genome Institute (http://genome.jgi-psf.org/Brafl1/Brafl1.home.html). The genome was first analyzed to find putative metallothionein genes that had already been annotated. Further homology searches were performed using BLAST and the NCBI and UNIPROT databases [20], applying one MT sequence from each of the 15 MT families in Kägi′s classification as the query (*cf*. www.bioc.unizh.ch/mtpage/classif.html). The *in silico* translated putative MT sequences were characterized by using the bioinformatic facilities of the EBI-SRS platform [21], the main objective being the identification of metal binding functional motifs by using the PROSITE algorithm [22]. Furthermore, a scan was made of the BLOCKS database to identify the most highly conserved regions of protein families contained in the identified sequences [23]. Finally, the Metal Detector (v1.0) software (http://metaldetector.dsi.unifi.it/v1.0/) [24] was used to predict the metal binding capacity of the Cys and His residues in the selected sequences, as well as the DIANNA web server [25] which also predicts the most likely bound metal. To search for *cis* gene expression control elements (MRE and ARE) and TATA boxes of the putative *MT* genes, a 1000-bp region upstream of their predicted transcription initiation site was screened using the transcription element search system (TESS), available online at http://www.cbil.upenn.edu/cgi-bin/tess/tess. Gene structure schemes were drawn with the gene structure display server [26] at http://gsds.cbi.pku.edu.cn/.

### Construction of amphioxus MT expression vectors

The coding sequences of the putative *MT* genes identified *in silico* were used to perform a BLAST search of the *Branchiostoma floridae* cDNA database (http://amphioxus.icob.sinica.edu.tw/) [27]. After analyzing all the retrieved ESTs and cDNAs, two clones including the full sequences encoding two non-homologous MTs were selected for recombinant expression: clone rbfeg037o01, for one *Branchiostoma floridae* MT sequence (*BfMT1*), and clone bfne062f22, including the second MT sequence (*BfMT2*). These clones were kindly provided by the Center for Genetic Resource Information at the Japanese National Institute of Genetics, in Mishima, Japan. The *BfMT1* and *BfMT2* coding regions were PCR-amplified from these clones, using as primers: BfMT1 upstream 5′-AAAGGATCCATGCCTGATCCCTGCAACTGTGCA-3′; BfMT1 downstream 5′-AAGCTCGAGTTACAGGTGAGCCGCTGGAG-3′; BfMT2 upstream 5′- AAAGGATCCATGCCAGACCCCTGTTGTGAG-3′; and BfMT2 downstream 5′- AAGCTCGAGTCACTTGCAGCAGTCACAACC-3′. This reaction introduced a *Bam*HI restriction site (underlined) before the ATG initiation codon, and a *XhoI* site (underlined) after the stop codon, of the BfMT coding regions. PCR conditions used were in accordance with the recommendations for the Expand High Fidelity PCR System (Roche) and the annealing step was performed at 55°C. The PCR products were isolated from 2% agarose gels, digested with *BamH*I*–Xho*I (New England Biolabs), and directionally inserted in the pGEX-4T-1 expression vector (GE Healthcare) for the synthesis of GST (glutathione-S-transferase) fusion proteins (*i.e*., GST-BfMT1 and GST-BfMT2). Ligations were performed using the TAKARA DNA ligation kit (v2.1) (Takara Shuzo Co), and the ligation mixtures were used to transform both *E. coli* DH5α and BL21 cells. The *GST-BfMT1* and *GST-BfMT2* constructs were automatically sequenced (Applied Biosystems Abiprism 310, PerkinElmer) using the BigDye terminator v3.1 kit (ABI Biosystems).

### Preparation of recombinant and *in vitro*-constituted metal-MT complexes

For recombinant synthesis of the metal-BfMT complexes, the pGEX–BfMT1 and pGEX–BfMT2 plasmids were transformed into the *E. coli* BL21 protease-deficient strain. Recombinant bacteria grown under metal supplemented conditions (300 µM ZnCl_2_, 300 µM CdCl_2_, or 500 µM CuSO_4_) and fusion protein purifications were carried out essentially as previously described [28], [29], [30]. At the end, the MT-containing fractions eluted from an FPLC Superdex 75 column (GE-Healthcare) in 50 mM Tris-HCl buffer pH 7.0, were pooled, aliquoted and stored at −80°C under argon atmosphere until required. *In vitro* metal-MT binding studies were performed through metal replacement and denaturation experiments. Titrations of the Zn-BfMT1 and Zn-BfMT2 preparations with Cd(II) or Cu(I) at pH 7 were performed as described earlier [28], [29], [31] using CdCl_2_ or [Cu(CH_3_CN)_4_]ClO_4_ solutions, respectively. The *in vitro* acidification/reneutralization experiments were adapted from [32]. Mainly, 10–20 µM preparations of the recombinantly synthesized metal-complexes were acidified from neutral (7.0) to acid pH (1.0) with HCl, kept at pH 1.0 for 20 min and subsequently reneutralized to pH 7.0 with NaOH. CD (circular dichroism) and UV–vis spectra were recorded at different pH throughout the acidification/reneutralization procedure, both immediately after acid or base addition, and 10 min later. During all experiments strict oxygen-free conditions were maintained by saturating the solutions with argon. All the *in vitro*-obtained metal-MT samples were analyzed following the same rationale as for the recombinant preparations.

### Characterization of the metal-MT complexes

Metal content (S, Zn, Cd and Cu) of the purified metal-MT complexes was analyzed by means of ICP-AES (inductively coupled plasma atomic emission spectroscopy) in a Polyscan 61E (Thermo Jarrell Ash) spectrometer, measuring S at 182.040 nm, Zn at 213.856 nm, Cd at 228.802 and Cu at 324.803 nm. Samples were treated as in [33], and were alternatively incubated in 1 M HCl at 65 °C for 15 min prior to measurements to eliminate possible traces of labile sulfide ions, as otherwise described in [34]. Protein concentration was calculated from the acid ICP-AES sulfur measure, assuming that in this case, all S atoms are contributed by the MT peptide. CD measurements were performed by using a Jasco spectropolarimeter (model J-715) interfaced to a computer (J700 software) maintaining a constant temperature of 25 °C using a Peltier PTC-351S apparatus. Electronic absorption measurements were performed on an HP-8453 Diode array UV–visible spectrophotometer. All spectra were recorded with 1–cm capped quartz cuvettes, corrected for the dilution effects and processed using the GRAMS 32 program.

### ESI-MS (electrospray ionization mass spectrometry) analyses of the metal-MT complexes

Electrospray ionization time-of-flight mass spectrometry (ESI-TOF MS) was applied to assess the molecular mass of the metal-BfMT1 and metal-BfMT2 complexes. The equipment used was a Micro Tof-Q instrument (Bruker) interfaced with a Series 1200 HPLC Agilent pump, equipped with an autosampler, which were controlled using the Compass Software. Calibration was attained with 0.2 g of NaI dissolved in 100 mL of a 1∶1 H_2_O:isopropanol mixture. Samples containing MT complexes with divalent metal ions were analyzed under the following conditions: 20 µL of protein solution injected through a PEEK (polyether heteroketone) column (1.5 m × 0.18 mm i.d.), at 40 µL min^−1^; capillary counter-electrode voltage 5 kV; desolvation temperature 90–110 °C; dry gas 6 L min^−1^; spectra collection range 800–2500 m/z. The carrier buffer was a 5∶95 mixture of acetonitrile:ammonium acetate/ammonia (15 mM, pH 7.0). Alternatively, the Cu-BfMT1 and Cu-BfMT2 samples were analyzed as follows: 20 µL of protein solution injected at 40 µL min^−1^; capillary counter-electrode voltage 3.5 kV; lens counter-electrode voltage 4 kV; dry temperature 80 °C; dry gas 6 L min^−1^. Here, the carrier was a 10∶90 mixture of acetonitrile:ammonium acetate, 15 mM, pH 7.0. For analysis of apo-BfMT1, apo-BfMT2, Cu-BfMT1 and Cu-BfMT2 preparations at acid pH, 20 µL of the corresponding samples were injected under the same conditions described previously, but using a 5∶95 mixture of acetonitrile:formic acid pH 2.4, as liquid carrier, which caused the complete demetalation of the peptides loaded with Zn(II) or Cd(II) but kept the Cu(I) ions bound to the protein.

### DEPC (diethyl pyrocarbonate) protein modification assays

Covalent modification experiments with DEPC were performed essentially as described in [35]. A fresh DEPC solution in absolute ethanol (DEPC:ethanol 1∶200 v/v) was allowed to react with a 100 µL solution of the metal-MT complexes (ranging from 0.2×10−4 to 2.1×10−4 M) in 50 mM Tris-HCl buffer, pH 7.0, for 20 min at room temperature. The resulting DEPC:protein ratios used were 7∶1 for Zn(II)- and Cd(II)-BfMT1. After incubation, all samples were immediately analyzed by ESI-TOF MS, under the conditions described above.

### Analysis of expression of amphioxus MT genes

To test the expression of both *BfMT1* and *BfMT2* genes, and to determine the main gene regulation features, some organisms of the European lancelet species (*Branchiostoma lanceolatum*) were kindly offered by Dr. Hector Escriva, from the *Observatoire Océanologique* in Banyuls-sur-Mer, France. For RNA purification, whole organisms were treated with the RNALater solution (Qiagen), and the total RNA was extracted by using the RNeasy Mini Kit (Qiagen), and cleansed from DNA contamination by DNAase digestion. Purified RNA was quantified and stored at −80°C until use. The Qiagen OneStep RT-PCR Kit was used for reverse transcription of 1 µg of total RNA, using the same specific oligonucleotides designed for cloning purposes. Final PCR products were sequenced as described previously. To semi-quantify the transcription rates of the *BfMT1* and *BfMT2* genes in *B. lanceolatum*, several of the sea-collected organisms were washed in sterile PBSx1 buffer and grouped for metal treatment: a solution of CdCl_2_ at 0.03 mM, 0.3 mM or 3 mM; or a solution of CuSO_4_ at 0.05 mM, 0.5 mM or 5 mM, final concentrations. A non-treated group was also maintained to obtain the corresponding control values. The corresponding metal salts were added onto the reconstituted Reef Crystals (Aquarium Systems) as synthetic sea water, and the treatment lasted for two days. After 48 h, all organisms were washed and total RNA was extracted as previously described. Semi quantitative RT-PCR was performed using the same RT-PCR Kit, on 30 ng of total RNA as template. Actin was used as conserved housekeeping gene to check for an equal retrotranscription rate among samples. PCR reactions were loaded onto a 1.5% agarose gel and bands were quantified by using the QuantityOne v.22 software from BioRad. Data are shown as the ratio of band intensities in relation to the corresponding actin. Statistical analysis was performed by using the GraphPad Prism 5 software (La Jolla, USA).

### Metal content measurements in amphioxus organisms

After incubation for two days in the metal-supplemented sea water medium described for the semi quantitative RT-PCR experiment, some organisms from each treatment condition were used to measure total body metal content. We evaluated not only total Cd and Cu contents, but also Zn and Fe, to ascertain possible metabolic relations. The organisms were washed with miliQ water, dried at 95°C, weighted, and finally decomposed in 1 ml of Trace Metal Grade (Fisher) nitric acid by heating at 80°C for 1 hour. After cooling to room temperature, samples were transferred to 15 ml ﬂasks, the volume was adjusted to 10.0 ml with Milli-Q water, and the total metal content of the samples measured by ICP-AES, as previously described for MT preparations. Control experiments without organisms were run in parallel to determine the background metal content in all the solutions used. All glassware and plasticware was acid-washed overnight with 10% nitric acid prior to use. Results are shown as total metal content normalized in relation to the dry weight of each organism. Statistical analysis was performed by using the GraphPad Prism 5 software (La Jolla, USA).

### Phylogenetic analysis

The set of MT sequences used to perform the phylogenetic analysis was selected by choosing one MT from each subfamily considered in the classification published in the Metallothionein Homepage (http://www.bioc.unizh.ch/mtpage/MT.html). Sequences were extracted from the UNIPROT website (http://www.uniprot.org/). Multiple sequence alignments were performed using the TCOFFEE web service at http://tcoffee.vital-it.ch/cgi-bin/Tcoffee/tcoffee_cgi/index.cgi [36], and stored in a *.PHY format, to be further used to construct the corresponding phylogenetic trees by the Maximum Likelihood algorithm (PHYLIP software package) [37]. Genetic distances were calculated using the Kimura 2-parameter method [38] and statistical support for nodes on the tree was evaluated using bootstrapping (1000 replications) [39].

## Results and Discussion

### The amphioxus genome includes two linked genes coding for rather dissimilar metallothionein isoforms: BfMT1 and BfMT2

The estimated size of the genome of *Branchiostoma floridae* is approximately 575 Mb contained in 19 chromosomes. Although it has been completely sequenced and annotated [16], the assignment of contigs to the physical chromosome map is not yet finished, which introduces some uncertainty to the studies of gene identification and localization in this organism. The examination of annotated *B. floridae* genes revealed only one sequence encoding a putative MT protein (ID 129939), hereinafter called BfMT1, already reported in a recent study on tunicate MTs [19]. Additionally a totally unpredicted ORF, coding for a second putative MT isoform (called BfMT2), was further identified after several homology searches (BLAST) using the cDNAs of members of different MT families as queries [5]. The predicted BfMT1 and BfMT2 protein sequences (Figure 1A) coincide with the general features of MTs, *i.e.* short, low molecular weight and cysteine-rich peptides, containing the PROSITE Cys-rich profile (ID PS50311). When submitting the BfMT1 sequence to a BLOCKS analysis, the signatures of the mollusk MT (PR00875), vertebrate MT (PR00860), and Diptera (*Drosophila*) MT (PR00872) families were identified (*E-value<3.6e-05*). On the other hand, the signature of crustacean MTs (PR00858) (with an *E-value  = 6.2e-05*), as well as other less significant MT signatures, were identified in the BfMT2 sequence. Finally, the Metal Detector algorithm predicted that 60% of cysteines in BfMT1 and 42.1% in BfMT2 sequences were able to bind metals with a probability higher than 0.6, these results agreeing with those obtained by the DIANNA web server for the prediction of metal binding sites. Before continuing our study, we decided to test whether the two BfMT1 and BfMT2 ORFs were indeed *real genes*, by checking the presence of the respective transcripts in lancelet organisms. To this end, we used the European *Branchiostoma lanceolatum* species, which is available from the near Mediterranean coast. Hence, RT-PCR assays were performed on total mRNA isolated from recently-captured organisms, by using isoform specific primers. A single band was obtained in each case, corresponding to the expected size deduced from the position of the primers in the *BfMT1* and *BfMT2* cDNA sequences (255 bp and 150 bp respectively, Figure 1B). This showed that both ORFs were transcribed to mature mRNAs in *B. lanceolatum*, and therefore that both *BfMT1* and *BfMT2* can be considered as functional genes. The corresponding PCR products were purified, cloned and sequenced. Their sequences fully matched those of the cDNA initially retrieved from the *in silico* searches in the *B. floridae* data bank, and thus these results also served to verify the identity of the BfMT1 and BfMT2 protein sequence between the two *Branchiostoma* species. While this manuscript was under revision, the *B. lanceolatum* transcriptome first release, obtained by shotgun assembly, was published [40]. In Fig. S1, we include the sequence of the BLAST retrieved clones, putatively correspondent to BlMT1 and BlMT2 cDNAs in this lancelet species.

### The amphioxus MT genes exhibit different structure and transcript processing patterns, which include alternative splicing for BfMT2

The location of the two *BfMT* genes in the amphioxus genome indicates a linked position in the *B. floridae* genome, in the negative strand of scaffold #398. Exact locations are [312336–314030] for *BfMT1* and [400166–403098] for BfMT2 (Figure 2). The identification of the complete *BfMT2* structure was only possible when working with the NCBI genome data, since in the original amphioxus genome platform (DOE Joint Genome Institute), almost all the corresponding region was of undetermined sequence, showing strong evidences of the presence of transposable elements, which are extremely common in amphioxus [17]. This situation may explain why this gene has remained undetected until now. The full sequence of both genes, as well as that of the flanking regions, is shown in Figure 3. BfMT1, expanding through almost 1.7 kpb in the genome, is composed of 4 exons separated by 3 introns, of 653−, 414−, and 372-bp. The first and last exons comprise respective 5′ and 3′ UTRs of regular length. Significantly, a considerable number of transcription factor binding elements could be identified *in silico*, comprising putative metal response elements (MRE), antioxidant response elements (ARE) and TATA box, which predict a gene regulation pattern similar to that of vertebrate *MT* genes. *BfMT2* also has a 4-exon/3-intron structure, but since the *BfMT2* introns are considerably longer (862 bp, 1249 bp and 615 bp) than those of *BfMT1,* the entire gene is considerably more extended in the genome. Most surprising, however, was the observation that the *BfMT2* cDNA sequence retrieved from the amphioxus data bank (bfne062f22 clon) did not correspond to the *canonical* cDNA expected from the splicing of the three *BfMT2* introns, but was the result of an alternate splicing pattern that skips exon 2 (*cf.* Figure 4). Only some small discrepancies between the sequence of the bfne062f22 cDNA and that of the predicted [exon1-exon3-exon4] sequence were observed, which are perfectly accountable due to natural variability between the different lancelet populations used as sources for these data. In fact, a thorough search of the *B. floridae* genome failed to identify any other possible coding region for BfMT2. An analysis of the presence of clones corresponding to the long mRNA in the cDNA databank readily suggests it can be considered an extremely rare transcript, since only a single clon (#rbfad023g19) out of the total of 132066 retrieved clones. This is concordant with the fact that no PCR product was amplified in the RT-PCR assay shown in Figure 1, which was performed by using primers corresponding to exons 1 and 4. Conversely, the number of clones for the short BfMT2 mRNA (that corresponding to bfne062f22), amounted to 90, being mostly abundant in the egg and adult stages (24 and 32, respectively). Overall, it seems plausible to assume that this short transcript, encoding for an MT peptide, is the most prominent product of the BfMT2 gene. Several MREs and AREs, but no canonical TATA box, could also be predicted in the 5′ *BfMT2* promoter region, as also shown in Figure 2. To support the functionality of these MREs, the *B. floridae* genome platform was searched for the presence of a putative MTF-1 coding gene, which we were able to locate in scaffold #418, using as a query the sequence of the zebra fish MTF-1 (*D. rerio).* The corresponding product, ID 155422, is annotated as a nucleic acid (MRE), zinc ion binding protein including the corresponding zinc-finger motifs, to be considered a canonical MTF-1 transcription factor.

### The two amphioxus MT genes are distinctly expressed at non-induced conditions and they are distinctly regulated by cadmium and copper ions

To investigate the response of both amphioxus MT genes to metal exposure, several organisms of the *B. lanceolatum* species were treated with different concentrations either of CdCl_2_ or CuSO_4_. As there was no previous reference for metal treatment for amphioxus, three metal concentrations ranging between two orders of magnitude (0.03-to-3 mM Cd and 0.05-to-5 mM Cu) were first assayed in order to determine sub-lethal conditions. All organisms kept at the highest metal concentration died within one day, and therefore *MT* transcriptional activity data were evaluated by semi-quantitative PCR from the other conditions, as well as from non-treated animals for basal expression information. *BlMT1* and *BlMT2* transcription patterns are already different in the absence of metal supplementation, since while *BlMT1* shows a significant constitutive expression rate, *BlMT2* transcripts are almost undetectable (Figure 5). Cadmium is a very good *BlMT1* inducer, since at both concentrations assayed (30 and 300 µM) the mRNA abundance approximately doubles that of the control conditions. But *BlMT2* is even more responsive to Cd, because this metal ion provokes, at 30 µM, an eight fold increase in the gene transcription rate in comparison with the control group (P<0.1) and threefold for Cd 300 mM (P<0.5). Treatment with copper did not significantly increase the number of *BlMT1* transcripts (P>0.5) at either of the two tested concentrations (Figure 5), while surprisingly, it proved to be a very strong inducer of *BlMT2*, specially in 500 µM Cu-treated organisms, where a 12 fold increase was observed in comparison with the background expression (P<0.01). Overall, it is clear that in physiological conditions, the *BlMT1* gene is constitutively transcribed, in contrast to *BlMT2,* which remains practically silent. Cadmium treatment induces both gene expressions, although at a much more pronounced rate for *BlMT2*. The less pronounced induction capacity, or even repression effect, of high cadmium concentrations has been observed before in metal response studies carried out with other model organisms, such as that for *C. elegans* MT genes [41], and is attributable to the inhibitory transcription activity of Cd (II) ions [42]. Copper has little effect on *BlMT1* transcription, but in contrast, it provokes a significant increase in *BlMT2* mRNA accumulation. However, this trend can be related to the BlMT2 peptide copper-binding incapacity, as discussed later in this study. In this scenario, the unbound Cu(I) ions would remain free in the organism and therefore continue to induce gene expression, through the identified MRE elements. The experimentally observed response to metal induction for both BfMT genes fully agrees with the functionality of the multiple MREs identified in the promoter regions of the respective genes, as reported in the *in silico* analysis section (*cf*. Figure 3).

### The amphioxus metal homeostasis is disrupted by cadmium and copper overload in a different way

Metal accumulation was measured in lancelets exposed (or non-exposed) to Cd or Cu in the same experimental conditions as for the transcriptional pattern studies reported in the previous section. Besides Cu and Cd, Fe and Zn contents were also measured to assess whether there was any significant interrelation between the pathways ensuring the homeostasis of these metals, as established in relevant model organisms, such as yeast [43]. All the results are summarized in Figure 6. Cadmium accumulation was readily evident in Cd-intoxicated animals, reaching maximum concentrations of 71.26 µM Cd/g dry weigh after 30 µM Cd treatment, and even 1373.99 µM Cd/g dry weigh for 300-µM Cd doses. As expected, no cadmium was accumulated in control or in Cu-treated organisms. These results are indicative of a notable disruption of the Cd homeostasis mechanisms in high Cd conditions, which is in concordance with the *MT* gene repressor effect described in the preceding section, and with a parallel phenomenon reported for MT-null *C. elegans* mutants [44]. Significantly, 300 µM Cd also deregulates internal Zn and Fe levels, which increase exponentially (P<0.01) in comparison to the non-treated organisms, while at a lower Cd dose (30 µM), there is no significant alteration of any physiological metal content (Zn, Cu. Fe). This leads to the hypothesis that the decrease in both MT gene expression levels would severely impair global metal homeostasis under heavy Cd intoxication conditions. On the other hand, Cu overload, at any concentration, has a poor effect on Zn and Fe contents, which remain as in control animals. Conversely, the internal pools for copper are significantly higher, in this case also with a much more drastic accumulation behavior also at the lower assayed concentration (50 µM).

### The recombinant synthesis of the BfMT1 and BfMT2 proteins

DNA sequencing of the BfMT1 and BfMT2 coding segments in the pGEX expression constructs ruled out the presence of any artifactual nucleotide substitution. Furthermore, SDS-PAGE analyses of total protein extracts from the transformed BL21 cells showed the presence of bands corresponding to the expected GST-BfMT1 and GST-BfMT2 sizes (data not shown). Homogeneous metal-BfMT1 and metal-BfMT2 preparations were obtained from 5-L *E. coli* cultures at final concentrations ranging from 2.6×10^−4^ M for divalent metal ions to 0.2×10^−4^ M for Cu complexes. Acidification of the Zn-BfMT complexes yielded the corresponding apo-forms, with molecular masses of 8476.2 Da for BfMT1 and 5053.0 Da for BfMT2, fully concordant with the calculated average theoretical values (8476.7 Da and 5053.9 Da, respectively) for the synthesized products. This confirmed both the identity and purity of the recombinant polypeptides.

### The Zn-binding abilities of BfMT1 and BfMT2 points to an intermediate Zn-/Cu-thionein character for both isoforms

The recombinant synthesis of BfMT1 and BfMT2 in Zn-supplemented *E.coli* cultures rendered a mixture of Zn-complexes of different stoichiometries (Figure 7). For BfMT1, the major species was Zn_7_-BfMT1, with the significant presence of lower amounts of the Zn_6_- and Zn_5_-BfMT1 complexes. Conversely, for the BfMT2 isoform, a major Zn_6_-BfMT2 species was accompanied by minor equimolar Zn_5_- and Zn_4_-BfMT2 complexes. These results match well the mean Zn-per-protein content calculated from ICP-AES measurements (Table 1), and are in accordance with the number of putative coordinating residues in their peptide sequences: 19 Cys and 2 His in BfMT1 and 18 Cys in BfMT2 (*cf*. Figure 1). The CD spectra of both preparations were practically silent in the corresponding spectral region (*ca*. 240 nm for Zn-Cys chromophores), this suggesting a poor folding degree of the clusters formed about the metal ions (Figure 7). Interestingly, the ESI-MS spectrum of the Zn-BfMT1 preparation revealed the presence of very minor peaks, which could be unambiguously identified as resulting from a BfMT1 truncated form, after the loss of the eight C-terminal protein residues (*i.e.* TTPPAAHL). Curiously, the corresponding Zn-complexes exhibit the same stoichiometries (Zn_7_-_,_ Zn_6_- and Zn_5_-) as those of the whole peptide, which indicates that this minor posttranslational event does not affect the coordinating capacity of BfMT1, and indirectly suggests that the His residue in the penultimate position does not participate in zinc binding. Histidine participation in BfMT1 metal coordination was also ruled out through DEPC-modification experiments, which indicated that neither of the two His residues present in BfMT1 was involved in Zn-binding (data not shown).

Concerning the observed minor truncation of BfMT1, it is noteworthy that a similar event has been observed for *C. elegans* CeMT1, produced under equivalent conditions. These two peptides have in common a C-terminal region devoid of cysteines, and with a similar amino acid composition. Interestingly a revision of our previous CeMT1 MS data [35] revealed a truncation of the three final residues of its metal-complexes, this suggesting the susceptibility of these free peptide tails to endoproteolysis.

### The Cd-binding abilities of BfMT1 and BfMT2 indicates a preference of BfMT2 over BfMT1 for cadmium dealing

The biosynthesis of the BfMT1 and BfMT2 isoforms in Cd-supplemented bacterial cultures revealed much more significant differences between their metal binding properties. Hence, BfMT1 rendered a mixture of many species, the two most abundant being, in this order, Cd_8_-, Cd_9_S- and Cd_7_-BfMT1 in this order (Figure 7). Among the minor peaks, many species of high Cd content and containing sulfide ligands could be envisaged. The important presence of S^2−^-containing complexes in this sample was confirmed by two independent results: first, the discrepancy between the Cd-per-MT contents measured by normal and acid ICP-AES results (Table 1); and second, the CD spectra of the Cd-BfMT1 preparation exhibiting the typical absorptions due to the Cd-S^2−^ bonds at *ca.* 300 nm, besides a broad signal centered at 250–260 nm contributed by the Cd(SCys)_4_ chromophores [34]. In marked contrast with these results, the folding of the BfMT2 peptide ensuing Cd coordination practically yielded a single Cd_6_-BfMT2 species. Only very minor species could be detected, corresponding to Cd_7_- and Cd_6_S_2_-BfMT2 (Figure 7). The latter would account for the only slight divergence between normal and acid ICP measurements) and the fact that the CD spectrum showed the typical fingerprint attributable to tetrahedral Cd(SCys)_4_ chromophore absorbing at *ca*. 250 nm, and only a minor absorption at (-) 275 nm. The presence of S^2−^-containing complexes in the preparation of both Cd-BfMT1 and Cd-BfMT2 preparations prompted us to analyzed whether their CD fingerprints evolved over time, a phenomenon that we have previously reported for preparations of the yeast Cd-Cup1, containing a significant proportion of S^2−^-containing complexes [45]. The main changes are observed for the Cd-BfMT1 CD fingerprint, in which the initial broad signal evolves to form two new narrower signals *ca.* 250 and 275 nm while maintaining the absorption at *ca.* 300 nm (contributed by the Cd-S^2−^ chromophores) (Figure 8D). Interestingly, while the more important CD variations are associated to the Cd-BfMT1 preparation, the more important speciation changes over time are observed for Cd-BfMT2. The former, which starts from a mixture of species with a major full Cd_8_-BfMT1 complex, ends in a truncated Cd_8_S-BfMT1 form. On the contrary, the single Cd_6_-BfMT2 species ends as a mixture of sulfide-containing cadmium complexes of the protein (Figure 8D).

*In vitro* Cd-binding behavior of the BfMT isoforms was analyzed by two different approaches: (i) Cd(II) titration of the Zn-BfMT preparations, and (ii) acidification plus subsequent reneutralization of the recombinant Cd-BfMT samples. The Cd(II) titration of Zn-BfMT1 shows a point of saturation in the UV-vis, CD and ESI-MS spectra for 8 Cd^2+^ equivalents added (Figure 8A). Although further Cd^2+^ equivalent addition provokes losses in the intensity of CD and UV-vis spectra, the speciation obtained at this point remains constant, including a mixture of several Cd_x_- and Zn, Cd-BfMT1 species, with major Cd_8_-BfMT1, as revealed by the ESI-MS results (Figure 8B). As expected from the important contribution of the S^2-^-containing species to the recombinant Cd-BfMT1 preparation, its CD spectrum is not obtained at the end of the Zn(II)/Cd(II) replacement reaction, but the addition of several S^2−^ equivalents to the resulting mixture after the Cd(II) titration clearly indicated the incorporation of these ligands into the Cd-BfMT1 complexes, although neither the CD fingerprint nor the speciation of the recombinant preparation can be reproduced *in vitro*. When the recombinant Cd-BfMT1 preparation was acidified, reneutralized and several S^2−^ eq were added, a CD fingerprint and a fairly similar speciation to that of the initial sample was obtained (Figure 8C). All these results highlight the importance of the S^2−^ ligands in the formation of an important subset of Cd-BfMT1 complexes under *in vivo* conditions.

In all the steps of the Zn(II)/Cd(II) replacement reaction in the Zn-BfMT2 preparation (Figure 9A), the species obtained correspond to a mixture of Cd_6_-, Cd_7_- and Cd_8_-BfMT2 (Figure 9B). The CD spectra show an absorption *ca.* 245 nm for 4 eq of Cd^2+^ added and after the addition of more equivalents this signal fades to eventually render an absorption at *ca* (-) 250 nm. Neither the CD nor the ESI-MS results at any point of the titration reproduced the features of the *in vivo* Cd-BfMT2 preparations. Similar results to those achieved after the acidification, reneutralization and sulfide addition to the Cd-BfMT1 preparations were achieved for those of BfMT2. Figure 9C shows the CD spectra of the initial recombinant Cd-BfMT2 sample together with that obtained after reneutralization and how the addition of S^2−^ anions allows restoration of the initial CD fingerprint. ESI-MS data (not shown) also indicates that the initial speciation is recovered after S^2−^ addition.

Overall results obtained for cadmium-binding converge in the classification of BfMT1 as an MT poorly competent peptide for Cd(II) coordination, as its recombinant synthesis by Cd-supplemented *E. coli* cells yields a mixture of species, including a subpopulation of complexes with sulfide ligands. This behavior is accompanied by other features typical of MT peptides of Cu-thionein character [7], and mainly derived from the substantial presence of S^2−^ anions in the preparations, such as the evolution of the CD spectra over time, and the inability of the final stage of the Zn/Cd titration, and also of the denaturalization/renaturalization process, to reproduce the fingerprint and stoichiometry of the recombinant sample. Although BfMT2 is not completely free of these features, they are considerably less pronounced than for BfMT1, and it can therefore be assumed that BfMT2 is much closer to an optimized MT for divalent metal ion binding.

### Cu-binding abilities of BfMT1 and BfMT2: the essential inability of BfMT2 for copper binding

BfMT1 and BfMT2 recombinant synthesis in the presence of Cu(II) surplus was assayed under normal and low aeration conditions, since we previously showed how bacterial culture oxygenation can affect the metal content of the complexes produced in these conditions [30]. Although we invariably failed to recover any BfMT2 product from Cu-supplemented cultures, the Cu-BfMT1 syntheses rendered some interesting results that allowed characterization of the purified samples. In normal oxygenation conditions, BfMT1 yielded different types of preparations, namely Cu-preparations of type 1 and type 2. Both types were constituted by mixtures of Cu, Zn-BfMT1 species in which an M_9_- (Type 1) or M_10_-BfMT1 (Type 2) (M  =  Zn+Cu) were the major species, as shown by the ESI-MS analyses at pH 7 (Figure 7). Mass measurements at pH 2.4 revealed that the M_10_- complexes of type 2 were in fact mainly Cu_8_Zn_2_- species, in accordance with the ICP results (Table 1). On the contrary, a mixture of major Cu_4_Zn_5_-, and minor Cu_5_Zn_4_- plus Cu_6_Zn_3_-BfMT1 complexes, would account for the detected type 1 M_9_- aggregates. In contrast, low oxygenation conditions yielded a mixture of homometallic Cu-BfMT1 species ranging from Cu_9_- to Cu_12_-BfMT1 (Figure 7 and Table 1). Interestingly, regular oxygenation type 2 preparations render the most intense and well-defined CD spectra (Figure 7); the CD fingerprint of type 1 productions is also well defined but less intense, while that of the low-aerated productions is practically silent. This suggests that the presence of Zn(II) ions in type 1 and 2 preparations would be crucial for the building of a well-folded structure to the corresponding complexes.

The *in vitro* Zn(II)/Cu(I) replacement studies in Zn-BfMT1 (Figure 10A) afforded the typical CD signals observed when Cu(I) binds to MTs [30], [46]. Interestingly, at different stages of this Zn/Cu replacement process, CD fingerprints and speciations analogous to those obtained in the *in vivo* productions are obtained. Hence, the addition of 4 and 6 Cu(I) eq gives rise to a mixture of species in which M_9_- is the major one, like in type 1 productions, although both samples do not share the same CD fingerprint. Addition of 12 Cu(I) eq makes M_10_- the major species of a mixture that shows a CD envelope similar to that of the type 2 productions (Figure 10C). Finally, excess Cu(I) addition to this solution affords mixtures of homometallic Cu_7_- to Cu_15_ complexes, which are somewhat similar to the results for the low-oxygenation syntheses. This is exactly the same situation that we described for the Zn(II)/Cu(I) titration of the yeast Crs5 MT [30]. The fact is that different stages of the replacement reaction reproduce the results of different recombinant productions, which in turn correspond to different copper availably conditions. Therefore this behavior suggests a copper-binding peptide that can cope with different physiological conditions yielding equally stable complexes.

On the other hand, the *in vitro* replacement of Zn(II)/Cu(I) in Zn-BfMT2 revealed some clues for understanding the impossibility of recovering recombinant Cu-BfMT2 complexes from the corresponding *E. coli* cells grown in copper supplemented cultures. When successive Cu(I) equivalents were added to the Zn-BfMT2 preparation, the UV-vis and ESI-MS spectra recorded at different steps unequivocally showed that Cu(I) was being bound to the peptide at increasing ratios, but the CD fingerprint remained practically silent (data not shown), which suggested a complete lack of structured folding. Significantly, exposure of the Zn(II)-BfMT2 complexes to Cu(I) caused the progressive generation of a highly complex mixture of heteronuclear (Zn(II), Cu(I)) and homonuclear (Cu(I)) species, also comprising apo-BfMT2, which evolved into the major form from 18 eq Cu(I) added on. Thus, it is reasonable to assume that the failure to reach a minimally folded state when coordinating Cu(I) ions leads to complete proteolysis inside the bacteria producing cells, a phenomenon that we have reported before for the mollusc *Megathura crenulata* [47] and the *Drosophila* MtnE MT isoforms [48], although only for the attempts to synthesize them from low-aerated (i.e. high intracellular copper) Cu-enriched cultures.

As this point, and admitting the obvious fact that BfMT2 is unsuitable as Cu-thionein, it is worth considering that BfMT1, although far from being optimal for Cu(I) coordination, presents the typical features of a partial Cu-thionein MT [7], since it is able to render homometallic Cu-complexes when recombinantly synthesized in highly Cu-enriched cells (*i.e.* cultured in Cu-supplemented media at low aeration), this adding up to its poor Cd-binding character, as discussed earlier.

### Phylogenetic and evolutionary studies places amphioxus MTs in the same echinoderm branch

Homology searches by performing BLAST in the UNIPROT database clearly show that both BfMT1 and BfMT2 protein sequences (sharing only 26.2% identity) clearly differ in sequence to other MTs, not being directly alignable to the vertebrate isoform peptides. Therefore there is a no clear lineage connecting any cephalochordate MT with the vertebrate MT prototype sequence. This is in concordance with the reported observation that most of the gene copies emerging from the large scale genome duplications on the vertebrate stem were lost, so that the relationship between non-vertebrate and vertebrate genomes is not always straightforward [16]. A protein distance similarity tree (Figure 11) shows the different positions of both amphioxus MTs, while revealing further interesting evolutionary relationships. First, it is worth noting that both cephalochordate (*Branchiostoma*) MTs are much more distant, and appear in a separate tree branch than the two tunicate (*Ciona* and *Hermania*) MTs. Interestingly, the tunicate MTs are situated in the “vertebrate branch”, close to the MTs of some fish (*Ictalurus* and *Scyliorhinus*), the most primitive vertebrates. Contrarily, the amphioxus MT group with the sea urchin isoform, at a greater distance of the vertebrate counterparts. This is in major concordance with the alternative hypothesis of a close relationship between amphioxus and echinoderms, leaving the tunicates close to vertebrates [18], which was proposed some years ago as opposed to the classic view that placed the Cephalochordates as close precursors of vertebrates.

## Conclusions

At least two genes encoding metallothionein peptides, here named *BfMT1* and *BfMT2*, are present, in tandem disposition, in the *B. floridae* genome. They have similar gene structures (4 exons interrupted by 3 exons) and exhibit an equivalent number of expression response elements (MREs and AREs), in their upstream DNA region. One gene encoding a putative MTF-1 has also been identified in the genome that is very similar to the MTF1 sequence in fishes, which supports the putative functionality of these MREs. ESTs and cDNA corresponding to both *BfMT* genes were retrieved from the *B. floridae* data banks, and RT-PCR experiments revealed that both transcripts were also present in the *B. lanceolatum* species, sharing a complete sequence identity. Surprisingly, both genes follow rather different expression strategies and patterns. *BfMT1* cDNA is composed of the four exon sequences; while *BfMT2* cDNA results from an alternate splicing that skips exon 2. The full length *BfMT2* cDNA would also be present in lancelets, but in extremely small amounts. *BlMT1* and *BlMT2* mRNA accumulation rates under Cd and Cu exposure also reveal very different metal responses: *BlMT1* is constitutively transcribed at considerable levels, and in control conditions, *BlMT2* is practically silent. Although metal treatment induces an enhanced synthesis of both isoforms, the relative response of *BlMT2* is much higher than that of *BfMT1*, and therefore while *BlMT1* can be considered an essentially constitutive gene, *BlMT2* is clearly inducible. The consideration of the Cd and Cu transcription regulation trends for the two genes, as well as the metal binding behavior of both recombinantly synthesized peptides converge in the hypothesis that they serve very different purposes in the amphioxus organisms. BfMT1 is a polyvalent metal binding peptide, able to coordinate any of the studied metal ions (Zn, Cd or Cu), rendering complexes stable enough to endure in physiological environments, which is fully concordant with the constitutive character of their encoding gene, and therefore, with a metal homeostasis housekeeping role. On the contrary, BfMT2 exhibits a clear ability to coordinate Cd(II) ions, while it is absolutely unable to fold into stable complexes under Cu(I) surplus, even as mixed Zn, Cu species. This is compatible with an essential cadmium detoxification role, which is consequently only induced in emergency situations. Notably, the Cd and Cu internal accumulation in metal treated organisms fully confirms this metal binding behavior, as discussed in the corresponding result sections. It is sensible to presume that there may be gene expression and/or metal binding differences between MT homologous isoforms of different lancelet species (*B. floridae* vs. *B. lanceolatum*). But it is worth noting that in the cases of MT polymorphic systems belonging to close species that we have previously analyzed, *e.g.* the pulmonate snails MTs, there are significant differences between the features of the several isoforms encoded in a same genome (paralogs), but MT isoforms of the same type (ortologs) exhibit extremely equivalent properties [49], [50]. This will presumably be the same case in the two lancelet species.

The key question of the metal specificity of different MT isoforms has been analyzed in depth in several model organisms exhibiting polymorphic MT systems. Molluscs (pulmonate gastropoda) and nematodes (*C. elegans*) are optimal for comparative purposes (Figure 11). Other interesting taxa, with somewhat opposing MT systems in terms of metal preferences, are vertebrates and the baker′s yeast, also included in the tree shown in Figure 11. Different situations are encountered in these groups. In the *Helix pomatia* [49] and *Cantareus aspersus* [50] snails, there are three MT isoforms, one with a polyvalent behavior, and the other two having evolved into extreme metal specificities: one highly inducible, for Cd detoxification, and the other rather constitutive, for Cu metabolism mainly linked to the needs of their hemocyanin respiratory pigment. Neither of the two reported MTs in the nematode *C. elegans* exhibits a Cu-thionein character, but the two CeMT peptides still exhibit different metal binding behaviors: being CeMT1, encoded from a somewhat constitutive gene and showing better Zn(II) binding abilities, being mostly associated to a housekeeping metal metabolism role; while a detoxification function is attributed to CeMT2, which is mainly synthesized after Cd induction, and which in turn shows better coordination behavior for this metal ion [35], [44].

None of the four MT isoforms present in mammals exhibits a clear Cu-thionein character, although the most putatively primitive, MT4, is clearly more prone to Cu-binding that the others [46]. Finally, the two highly dissimilar yeast MTs (Cup1 and Crs5) are of Cu-thionein character, but the extreme specificity for copper binding of Cup1 [45] is changed to a more subtle preference for Crs5 [30], compatible with a mixed metal metabolism role. A fairly comprehensive picture of MT differentiation in different taxa seems to emerge from these considerations. On the one hand, there are MT isoforms with somewhat polyvalent metal binding abilities, able to form physiologically stable complexes with all kinds of the considered metal ions (mainly the physiological Zn and Cu), without exhibiting any clear metal preference, and which are encoded in a constitutive, or poorly inducible, pattern. These isoforms always appear at the root of the branches in phylogenetic or protein distance trees (*cf*. Figure 11), and are best exemplified by yeast Crs5, snail Cd/Cu MTs, *C. elegans* MT1 or vertebrate MT4. Then, in each group of organisms, highly specialized MT isoforms, optimized for the handling of a specific metal ion, would have evolved to fulfill specific metabolic needs or cope with intoxication threads, according to the specific conditions of their habitats. On the one hand, metal-responsive MTs would have emerged as defense peptides against metal surplus, synthesized from clear metal-inducible genes, as found for the Cd-MT snail isoforms and the Cup1 yeast MT. On the other hand, MT specific isoforms related to special basic metabolic needs would have remained constitutive, as is the case with the Cu-MT forms in snails. This kind of specialization pattern would easily account for the tremendous heterogeneity among metallothionein peptides [4], as each differentiation event would have been highly specific and independent in each branch of the tree of life, and starting from different *substrate* peptides. However, since the main determinant of MT metal specificity has been identified as its protein sequence, this including both the number and position of the coordinating residues (mainly Cys) and also the nature of the intercalating amino acids [49], it is not surprising that similar amino acid motifs are detected for MT peptides converging in a preference for the same metal ion, as has been long reported [51]. And this is also precisely what we observe for the two characterized BfMT isoforms that lancelets synthesize: an MT isoform for general metal handling (BfMT1), and a specialized form devoted to metal (eventually cadmium) defense (BfMT2). Precisely, BfMT1 appears in a position in the tree in Figure 11 that is closer to the common central link of all MTs, while the most specialized BfMT2 is in a longer (therefore distant) branch, which is concordant with the above proposed evolutionary scenario.

## Supporting Information

Figure S1 **Sequences of the** ***B*** *.* ***lanceolatum*** **BlMT1 and BlMT2 cDNAs.** This data were deposited in the NCBI Transcriptome Shotgun Assembly (TSA) database, as reported while the current manuscript was under revision [40].
(DOC)
