# Supplementary material for: Metal Dealing at the Origin of the Chordata Phylum: The Metallothionein System and Metal Overload Response in Amphioxus
Source: PLoS One. 2012 Aug 14;7(8):e43299. doi: 10.1371/journal.pone.0043299 (PMC3419175; doi:10.1371/journal.pone.0043299)
Supplement: Figure S1 — Sequences of the B . lanceolatum BlMT1 and BlMT2 cDNAs. This data were deposited in the NCBI Transcriptome Shotgun Assembly (TSA) database, as reported while the current manuscript was under revision [40]. (DOC) [file pone.0043299.s001.doc]

**Supplementary Figure S1.**

The *B. lanceolatum* transcriptome (TSA assembly, NCBI Bioproject PRJNA82409 [40]) was searched with BLAST for BfMT1 and BfMT2 homologous cDNAs. The JT862963 (A) and JT862963 (B) transcripts were respectively identified as putatively coding for BlMT1- and BlMT2-like (long version) proteins. Similarity guarantees the homology between the two isoforms in the two species, but heterogeneity in the information provided by different genome and EST projects (as observed for the *B. floridae*), isoform multiplicity and above all, the complexity of the amphioxus MT genes and coding regions renders the information presented below as definitely provisional, in the absence the *B. lanceolatum* genome sequence.

**(A)**

JT862963 GTATGCCTGATCCCTGCAACTGTGCTCAGAGCGGTGCGTGCTCCTGTAACGGACTGTGCC 60

BfMT1 --ATGCCTGATCCCTGCAACTGTGCACAGAGCGGAACGTGCTCCTGCGGCGGGCCGTGCC 58

***********************:********:.********** ..***.* *****

JT862963 AGTGCGGTGATGACTGCCAGTGTGGTGACGGCTGTAAATGTGTCGGCTGCAAACTTCACG 120

BfMT1 AGTGCGGCGATGACTGCCAGTGTGGTGACGGCTGTAAGTGTGTCGGCTGCAAACTTCACA 118

******* *****************************.*********************.

JT862963 GCAACGTCGACGTCGCTCTCACCTGTTGTGGCACCTGCACGGGGATAGGGAAGAACTGTG 180

BfMT1 GCAATGTCACTGACATTGTCACCTGCTGTGTTGACTGCAAGGGGATAGGGAAGAATTGTG 178

**** ***.. *:*. * ******* **** ..*****.*************** ****

JT862963 CCTGTGGATGCTCGTGTTGCCAGCCCGACGTTCCAGCCGTGACCGTGCTGACAACGCCTC 240

BfMT1 CCTGTGGCTGCTCGTGTTGTCAGCCTGATACTCCGGCGGTGGCCATTCTGACAACGCCTC 238

*******.*********** ***** ** . ***.** ***.**.* *************

JT862963 CAGCGGCTCACCTGTAAATTGCAGATCGTAAAACCCCATATAACATGGTCTGCCTTGTGG 300

BfMT1 CAGCGGCTCACCTGTAA------------------------------------------- 255

*****************

JT862963 AATGACGTCATGTAGTTACATTTAGTCATCTGATCGTTACATTGGAATTCATTATGTCAC 360

BfMT1 ------------------------------------------------------------

JT862963 CTGTTTCCCTGT 372

BfMT1 ------------

JT862963p MPDPCNCAQSGACSCNGLCQCGDDCQCGDGCKCVGCKLHGNVDVALTCCGTCTGIGKNCA 60

BfMT1 MPDPCNCAQSGTCSCGGPCQCGDDCQCGDGCKCVGCKLHSNVTDIVTCCVDCKGIGKNCA 60

***********:***.* *********************.** :*** *.*******

JT862963p CGCSCCQPDVPAVTVLTTPPAAHL 84

BfMT1 CGCSCCQPDTPAVAILTTPPAAHL 84

*********.***::*********

**(B)**

JT872034 GGCACAATCAGTATAAAGCATGGAGCACTGGACACGTCGTCATTTTCCTGCTACGCCACA 60

BfMT2_L ------------------------------------------------------------

JT872034 GCGCCCTTTGTGTTCGTGACTGTCCCAGACGCTACGAGAAAAGACTGACAAGATGCCAGA 120

BfMT2_L -------TTGTGTTCGTGACCGCCCAAGACACTACGAGGAAAGACTGACAAGATGCCAGA 53

************* * **.****.*******.*********************

JT872034 CCCCTGCTGCTCTGCCTGTACTGGATGCTCCACCTCCTGCAAGTCATGTAACTGTGACTG 180

BfMT2_L TCCCTGCTGTTCCGCTTGTGAGGGATGCTCCAGCACCTGTAACAAATGTAGCTGTGACTG 113

******** ** ** ***.. ********** *:**** ** :.*****.*********

JT872034 CTGCAAGTGTTGTGCCTCTTGCACTGGCTGCAGCCCC---AACTGCAACTCATGTGGCTG 237

BfMT2_L CTGCAAGTGTTGTGCCTCTTGCAAGGCCTGTGGTCCCGCAGCCGACTGCAACTGTGGCTG 173

***********************. * *** .* *** ..* .*:.*:..********

JT872034 TGACTGCTGCAAGTGCTGTGCCTCGTGTGACGGTTGCAAGTCTGGCTGCACCAGCTGTAG 297

BfMT2_L TGCCTGCTGCAAATGCTGTGCCTCTTGTGATGGATGCAAGTCTAGCTGCACCAGCTGTAG 233

**.*********.*********** ***** **:*********.****************

JT872034 CTGTGACTGCTGCAAATGAAGGGAAGCAG--ACAAGTACCACAG-------GAG-ACTGC 347

BfMT2_L CTGTGACTGCTGCAAGTAAGGAGAGACAATCACCAATACCACAGATCTATAGAGGACTGT 293

***************.*.*.*.**..**. **.*.******** *** ****

JT872034 AGCGGACTGGCCAGAATGGACATCAA------CAACACC--GTACATTCCAATACCTAGC 399

BfMT2_L GGCAGACTGGCTAGTATGGACATCAATGCTATCAGCACCTTGTACATTCCATTATCAAGC 353

.**.******* **:*********** **.**** **********:** *:***

JT872034 TGGCATGTTATGTTAG--AAGCTTAGCTTTTAATTAT-----------------CTTATA 440

BfMT2_L CGGAATGTTGTAAAAGCATAGCTTATGTTTCAGTGATGACGTCACAGAGGAAAGCATTTA 413

**.*****.*.::** :****** *** *.* ** *:*:**

JT872034 TATTACTAT-----TAGTGATACCTGCTACG--CAGTGAAGGATTG--ATGTCT------ 485

BfMT2_L AATGTCTATGGTGATAGCTAAGCTTGCATTTTTAAGTCTAAGTATCTTCTGTCTGTCCTT 473

:** :**** *** *:.* ***:: .*** :*.*::* .*****

JT872034 ------ATTGTGATGGCACAGCATGCATGCATTTTCAAGTTTAAGTATTTTTCTGTCCTT 539

BfMT2_L TTAGAATTTATGAATGGAAAAAGAGTAATCAGTATTAAAAAGATGTTTTATTCACATATT 533

:**.***: * *.*...:* *: ** *:* **.:: *:**:**:***: : .**

JT872034 ------TTAAAAATCATTTCTGAGATAAAGGAAATC--GGTGTTCAAAATGTTATT-CAG 590

BfMT2_L GAGTACTATCTACTTAAATGTAAAGCAGAAGATTTCTTGGAGATATAGAAATTATTGAAA 593

*::.:*.* *::* *.*.. *.*.**::** **:*:*.:*.*:.***** .*.

JT872034 ATGAATTCTAGAATTCTCAAATAT------ 614

BfMT2_L GTGCACTTTATTTTTTTAAAAAGTTGAATG 623

.**.* * ** ::** *.***:.*

JT872034p MPDPCCSACTGCSTSCKSCNCDCCKCCASCTGCSP--NCNSCGCDCCKCCASCDG-CKSG 57

BfMT2_L MPDPCCSACEGCSSTCNKCSCDCCKCCESCKACGPTVGCN-CGCACCKCCSSCVQTCKPG 59

********* ***::*:.*.******* **..*.* .** *** *****:** **.*

JT872034p CTSCS-CDCCK 67

BfMT2_L CTNCPGCDCCK 70

**.*. *****
